# EVALUATING IMPLEMENTATION FIDELITY TO A NURSE-LED CARE MODEL IN NURSING HOMES: A MIXED-METHODS STUDY

**DOI:** 10.1093/geroni/igac059.2026

**Published:** 2022-12-20

**Authors:** Raphaelle Ashley Guerbaai, Sabina De Geest, Michael Simon, Lori Popejoy, Nathalie Wellens, Kris Denhaerynck, Franziska Zúñiga

**Affiliations:** The Institute of Nursing Science, Basel, Basel-Stadt, Switzerland; Institute of Nursing Science, Basel, Basel-Stadt, Switzerland; The Institute of Nursing Science, Basel, Basel-Stadt, Switzerland; University of Missouri, Columbia, Missouri, United States; Haute Ecole La Source, Lausanne, Vaud, Switzerland; The Institute of Nursing Science, Basel, Basel-Stadt, Switzerland; University of Basel, Basel, Basel-Stadt, Switzerland

## Abstract

Implementation fidelity assesses the degree to which an intervention is delivered as intended. Little is known about how it acts as a moderator between an intervention and its intended outcome(s) and which factors affect the fidelity trajectory over time. We exemplify implementation fidelity in INTERCARE, a nurse-led care model implemented in eleven Swiss nursing homes (NH) successfully decreasing unplanned hospital transfers. A mixed-methods design was used, guided by the Conceptual Framework for Implementation Fidelity. Fidelity to INTERCARE’s core components was measured with 44 self-developed items at 4-time points (baseline, 6, 12 months after intervention start, 9 months post-intervention; fidelity scores were calculated for each component and overall. Structured notes from NH meetings were used to identify moderators affecting the fidelity trajectory over time. Generalized linear mixed models were computed to analyze the quantitative data. Deductive thematic analysis was used for the qualitative analysis. The quantitative and qualitative findings were integrated using triangulation. A higher overall fidelity score showed a decreasing rate of unplanned hospital transfers (OR: 0.65 (CI=0.43-0.99), p=0.047). Higher fidelity score to advance care planning was associated with lower unplanned transfers (OR= 0.24 (CI 0.13-0.44), p= < 0.001) and a lower fidelity score for communication tools (e.g., ISBAR) to higher rates in unplanned transfers (OR= 1.69 (CI 1.30-2.19), p= < 0.003). High implementation fidelity to INTERCARE was necessary to achieve a reduction in unplanned transfers. In-house physicians with a collaborative approach and staff’s perceived need for nurses working in extended roles were important factors for high fidelity.

